# Causes and circumstances of death in stimulant and opioid use—A comparative study

**DOI:** 10.1371/journal.pone.0297838

**Published:** 2024-02-07

**Authors:** Ada Åhman, Carl Johan Wingren, Anders Håkansson

**Affiliations:** 1 Department of Clinical Sciences Lund, Lund University, Psychiatry, Lund, Sweden; 2 Unit for Forensic Medicine, Department of Clinical Sciences Malmö, Lund University, Lund, Sweden; 3 Department of Forensic Medicine, Copenhagen University, Copenhagen, Denmark; University of Macerata: Universita degli Studi di Macerata, ITALY

## Abstract

**Aims:**

To investigate the individual characteristics, causes and circumstances around deaths in stimulant use, and to examine how individuals who died with stimulants in their body differ from individuals who died with opioids in their body.

**Methods:**

This study includes individuals who died during the years 2000–2018 and underwent a forensic autopsy at Forensic Medicine in Lund, Skåne County, Sweden. All individuals over 18 years of age with stimulants (n = 310), opioids (n = 2,039) or both stimulants and opioids (n = 385) in the body at the time of death, were included. The three groups were assessed regarding gender, age, place of death, BMI, other substances detected in forensic toxicological analysis, organ weights and underlying and contributing causes of death. The data were analysed by frequency and proportion calculations, cross-tabulations and comparisons of medians.

**Results:**

The median age at death of the study population (n = 2,734) was 45.5 years (interquartile range ☯IQR] 32–60 years) and 73.2% were men. The most common cause of death in the stimulant group was suicide (26.8%), higher proportion compared to the opioid group (20.8%) (p = 0.017) and in the polysubstance group accidental poisoning (38.2%), higher proportion compared to the opioid group (18.0%) (p<0.001). Death by transport accidents was significantly associated with the stimulant group (p<0.001) as well as death by other accidents (p = 0.016).

**Conclusions:**

Individuals who died with stimulants in their body died at a higher rate from suicide, transport accidents and other accidents, compared to individuals who died with opioids in their body. This study indicates the need to identify and prevent psychiatric conditions, elevated suicide risk, and risk-taking behaviors among people who use stimulants.

## Introduction

*Central nervous system stimulants* (stimulants) include widely used illicit drugs such as amphetamine, methamphetamine, cocaine, and 3,4-methylenedioxymethamphetamine (MDMA), as well as prescribed medications such as methylphenidate. The use of stimulants globally is extensive and seems to have increased during the last decade. The United Nations Office on Drugs and Crime estimated that 21.5 million people used cocaine and 36 million people used amphetamines during 2021 [1].

This group of substances have a stimulating effect on the central nervous system with symptoms such as elevated blood pressure, heart rate and body temperature [2]. The use of stimulants is associated with cardiovascular problems such as arrhythmias, acute myocardial infarction, cardiomyopathy [3–5] and cerebrovascular events such as stroke [6, 7]. The use is furthermore associated with numerous psychological effects such as paranoia, risk-taking behaviour, acute psychosis and suicide [2, 8, 9].

According to international statistics, Sweden is among the European countries with the highest drug-related mortality [10], although international comparisons are difficult to make due to different definitions of drug-related mortality, differences in rates of forensic examination [11], and different coverage of the official mortality statistics [12]. In Sweden, amphetamine has traditionally been the dominant substance among people who inject drugs, although in recent years a mixed use has increasingly taken on a more central role [13, 14].

Considering the continued high morbidity and mortality and the globally growing problem with stimulant use, there is an urgent need to understand more about stimulant users’ particular risks in order to be able to offer effective tailored preventive interventions to reduce the harmful effects of the use.

The evidence regarding mortality in stimulant users has increased in recent years. Stimulant users have a mortality rate 3–6 times that of nonusers [15, 16] and in the Swedish setting 4–8 times [17, 18]. While the causes of death among opioid users have been dominated by opioid overdoses [19], stimulant users have been found to die from polysubstance overdoses, cardio- and cerebrovascular disease, accidents and suicides [20]. Although the number of studies on mortality and causes of death among stimulant users has increased, there is a continued need for more studies with better quality. This particularly applies to the reporting of study details such as the exact manner in which cause of death is ascertained and the reporting of specific ICD code [16]. There is also a lack of data on comparisons between the deaths of stimulant users and the deaths of people using other substances [21].

In this study, we examined all deaths in the south of Sweden (here defined as the catchment area of the unit for forensic medicine in Lund, Sweden) between the years 2000–2018 that were the subject to a forensic autopsy and where stimulants or opioids were detected in the blood of the deceased. The purpose of the study was to 1) investigate the individual characteristics and circumstances around the deaths 2) examine the causes of death 3) examine how individuals who died with stimulants in their body differ from individuals who died with opioids in their body.

## Methods

### Study procedure

The research was approved by the Swedish Ethical Review Authority (file number 2019–04759). As the study involved exclusively deceased subjects, no informed consent procedure was carried out. The individuals in this study were retrospectively identified in the case registry of the Swedish National Board of Forensic Medicine, which covers all forensic autopsies in Sweden since 1994. The registry includes data from forensic medical reports such as causes and manner of death, autopsy findings such as toxicological analysis (illicit drugs as well as other identified substances) and circumstances surrounding the death. Data about the cases that were subjected to a forensic autopsy between 2000–2018 were collected in 2020 and processed in 2020–2021.

About 6% of the more than 90,000 deaths that occur annually in Sweden, are subject to forensic autopsy [22]. According to Swedish legislation, physicians should report deaths to the police whenever there is an obvious or suspected unnatural death or where the circumstances surrounding death are unclear. This includes deaths among persons with a known or suspected substance use disorder or where the death may have occurred because of external influences (such as intoxication). The police will in approximately 95% of these cases request a forensic autopsy [22]. The autopsy, together with a police report describing the circumstances surrounding the death, forms the basis for the forensic medical report, where the underlying and contributing causes of death are determined according to the International Statistical Classification of Diseases and Related Health Problems (ICD) [23].

### Inclusion and exclusion criteria

The study includes individuals who died during the years 2000–2018 and underwent a forensic autopsy at Forensic Medicine in Lund, Skåne County, Sweden. Individuals with age over 18 years from the same geographic area during the study period and with stimulants, opioids or both stimulants and opioids in the body (regardless of concentration) at the time of death, were included. The original data set contained 2,746 individuals. Twelve individuals were excluded due to lack of information on gender (n = 8), age (n = 3) and detected substances (n = 1). The final data set then contained 2,734 individuals.

### Study population and variables

The study population was divided into three groups:

1) The opioid group—Individuals with opioids detected in forensic toxicological analysis (and no detection of stimulants, however with a possible detection of other substances, such as benzodiazepines and THC). Opioids were defined as heroin (6-monoacetylmorphine), methadone, buprenorphine, morphine, oxycodone, fentanyl (and fentanyl analogues) or tramadol.

2) The stimulant group—Individuals who had stimulants in forensic chemistry analysis (and no presence of opioids, however with a possible presence of other substances, such as benzodiazepines and THC)–the stimulant group. Stimulants were defined as amphetamine, methamphetamine, derivatives of central stimulant drugs (lisdexamphetamine, methylphenidate, dexamphetamine sulfate, modafinil), MDMA and cocaine.

3) The polysubstance group—Individuals who had *both* opioids and stimulants in forensic chemistry analysis (and a possible presence of other substances, such as benzodiazepines and THC).

The three groups were assessed regarding following variables: gender, age, place of death, BMI, other detected in forensic toxicological analysis, organ weights and underlying and contributing causes of death. All variables are dichotomous with the variable values “yes” and “no”, unless otherwise reported.

The BMI variable was divided in to four categories defined as Underweight (BMI<18.50), Normal weight (BMI = ≥18.50 - <25), Overweight (BMI = ≥25.00 - <30) and Obesity (BMI = ≥30.00). The other substances, besides stimulants and opioids, found in forensic chemical analysis at the time of death and included in the study were: alcohol (only cases with a blood alcohol concentration of > 0.1‰ were included, to avoid the risk of including cases of alcohol being produced in the body after death [24], benzodiazepines (diazepam, clonazepam, lorazepam, midazolam, nitrazepam, flunitrazepam, oxazepam, alprazolam, triazolam), Z-drugs (zolpidem or zopiclone), gabapentin, pregabalin and THC. The material in the forensic chemistry analysis was limited to blood, urine, muscle or eye fluid. All toxicological analyses were performed at the same national laboratory and the methods used are shown in a S1 Table.

### Causes of death

The underlying cause of death was reported, unless otherwise stated. The vast majority of the individuals in the data set had a cause of death stated according to the Swedish version of the Ninth Revision of ICD (ICD-9) [25]. In 27 individuals, the cause of death was stated according to the Swedish version of the Tenth Revision of ICD (ICD-10) [26]. These individuals were assigned a corresponding cause of death according to ICD-9 after a review by the authors.

The Swedish version of ICD-9 holds the code “E859”, “accidents due to poisoning”, which does not exist in the international version of ICD-9. In our data set, 297 of individuals originally had the code “E859” as the underlying cause of death, however, this has been replaced with the best corresponding code in the international version of ICD-9—the code “E858”, “accidental poisoning by other drugs”.

Eight individuals had an unnatural cause of death (ICD-9 code in the range 800–999) as their underlying cause of death but lacked an E-code.

### Statistical analysis

The data were analysed by frequency and proportion calculations, cross-tabulations and comparisons of medians. Significance testing of group differences regarding variable distribution has been carried out using Pearson’s chi-square test and Fisher’s exact test. The Mann-Whitney U test was used to compare medians between groups. Opioids, the most common drug among the deceased, were set as the reference value for the pairwise comparisons between the substance groups. All analyses were performed in SPSS (IBM SPSS statistics version 27 and 28 for Mac OS). P-values below 0.05 was considered statistically significant.

## Results

### Characteristics, forensic and substance-related data

Table 1 presents the study population (n = 2,734) based on characteristics, substance-related and forensic data. Most of the individuals were found in the opioid group (74.6%), 11.3% in the stimulant group and 14.1% in the polysubstance group. A majority of the deceased were men (73.2%) and the median age at death was 45.5 years (interquartile range ☯IQR] 32–60 years). The most common place of death was private housing (51.6%). A majority of the deceased were overweight or obese (50.7%). The most common drug beyond opioids and stimulants was benzodiazepines, present in 47.9% of the deceased, and in second place alcohol > 0.1‰ (26.0%). THC was found in 14.7% of deaths.

**Table 1:** Characteristics, forensic and substance-related data among the study population.

	Total, n = 2734	Opioids (and no stimulants), n = 2039	Stimulants (and no opioids), n = 310	P-value	Polysubstance group (Stimulants + opioids), n = 385	P-value
Age at death, median years (IQR)^a^	45.5 (32–60)	50 (34–64)	40 (28.8–51)	<0.001*	35 (26–43)***	<0.001*
Male gender, % (n)	73.2 (2002)	69.8 (1423)	82.9 (257)	<0.001*	83.6 (322)***	<0.001*
						
Place of death, % (n)						
Hospital	13.4 (366)	14.3 (276)	13.4 (39)	0.675	13.8 (51)	0.801
Other health care facility	2.8 (76)	2.4 (47)	3.1 (9)	0.508	5.4 (20)	0.002*
Private housing	51.6 (1412)	57.3 (1105)	43.3 (126)	<0.001*	49.1 (181)	0.003*
Other/unknown, not in healthcare	26.8 (733)	26.0 (499)	40.2 (117)	<0.001*	31.7 (117)	0.021*
Missing^b^	5.4 (147)	5.5 (112)	6.1 (19)	-	4.2 (16)	-
BMI, % (n)						
Underweight	5.9 (160)	6.1 (123)	7.4 (22)	0.379	3.9 (15)	0.097
Normal weight	42.2 (1155)	40.3 (814)	53.5 (159)	<0.001*	47.6 (182)	0.007*
Overweight	31.6 (865)	33.2 (672)	28.0 (83)	0.069	28.8 (110)	0.089
Obesity	19.1 (521)	20.4 (413)	11.1 (33)	<0.001*	19.6 (75)	0.724
Missing^b^	1.2 (33)	0.8 (17)	4.2 (13)	-	0.8 (3)	-
						
Other substances, % (n)						
Alcohol (>0.1‰)^c^	26.0 (708)	27.1 (553)	29.0 (90)	0.482	17.0 (65)	<0.001*
Benzodiazepines	47.9 (1310)	46.9 (957)	34.2 (106)	<0.001*	64.2 (247)	<0.001*
Z-drugs	15.9 (436)	18.2 (372)	5.8 (18)	<0.001*	11.9 (46)	0.003*
Gabapentin, Pregabalin	9.2 (251)	8.6 (176)	3.2 (10)	0.001*	16.9 (65)	<0.001*
THC	14.7 (402)	10.6 (217)	25.8 (80)	<0.001*	27.3 (105)	<0.001*

Opioids were set as the reference group for Chi-square comparisons between the substance groups. Numbers are presented as percentages (absolute number) if otherwise is not stated.

* p-value of <0.05 considered statistically significant

^a^ Mann-Whitney U test used to compare medians between groups

^b^ Proportion missing relative to the total number of participants

^c^ Analyzes carried out exclusively on blood

In pairwise comparisons between the opioid group and the other two groups, a few significant differences emerged. Women were most commonly found in the opioid group in which 30.2% were female in comparison to 17.1% in the stimulant group and 16.4% in the polysubstance group (p<0.001 respectively). The stimulant group and the polysubstance group showed significantly lower median age at death when compared to the opioid group, 40 years (IQR 28.75–51) and 35 years (IQR 26–43) compared to 50 years (IQR 34–64) (p < 0.001 respectively).

The stimulant group showed significantly higher proportion of individuals with normal weight and significantly lower proportion of obese individuals, (p<0.001 respectively) in comparison to the opioid group. The stimulant group also showed a lower proportion of deaths occurring in a private housing, and a higher proportion of deaths occurring in other or unknown setting.

Significant differences between the groups were identified regarding the prevalence of all substances examined. Alcohol was present to a significantly lesser extent in the polysubstance group (p<0.001), benzodiazepines occurred at significantly lesser extent in the stimulant group and at significantly greater extent in the polysubstance group (p<0.001, respectively). Z-drugs occurred at a significant lesser extent in the stimulant group (p<0.001) as well as in the polysubstance group (p = 0.003). The presence of THC was significantly more common in the stimulant group (p<0.001) as well as in the polysubstance group (p<0.001), when compared with the opioid group.

### Unnatural and natural causes of death

Unnatural cause of death was significantly associated with the polysubstance group (p<0.001) (Table 2). The prevalence of death by accidental poisoning were significantly higher (p<0.001) in the polysubstance group (38.2%) compared to the opioid group (18.0%). Homicides were more common in both the stimulant group (3.5%, p = 0.004) and the polysubstance group (3.9%, p<0.001) when compared to the opioid group (1.2%). Death by injury with undetermined manner of death were conclusively less common in the stimulant group—13.5% compared to 26.7% in the opioid group (p<0.001). Death by transport accidents was significantly associated with the stimulant group (p<0.001) as well as death by other accidents (p = 0.016).

**Table 2:** Underlying causes of death according to ICD-9 with unnatural causes of death specified.

	Total, n = 2734	Opioids (and no stimulants), n = 2039	Stimulants (and no opioids), n = 310	P-value	Polysubstance group (Stimulants + opioids), n = 385	P-value
Natural causes of death, % (n)	22.5 (615)	25.4 (518)	21.0 (65)	0.092	8.3 (32)	<0.001*
Unnatural causes of death, % (n)	77.5 (2119)	74.6 (1521)	79.0 (245)	0.092	91.7 (353)	<0.001*
Transport accident (E800-E845)	4.2 (116)	2.8 (58)	13.5 (42)	<0.001*	4.2 (16)	0.170
Accidental poisoning (E850-E869)	20.4 (558)	18.0 (368)	13.9 (43)	0.071	38.2 (147)	<0.001*
Other accidents (E880-E928)	4.6 (125)	4.6 (93)	7.7 (24)	0.016*	2.1 (8)	0.025*
Suicide and self-inflicted injury (E950-E959)	19.6 (537)	20.8 (424)	26.8 (83)	0.017*	7.8 (30)	<0.001*
Homicide and injury purposely inflicted by other persons (E960-E969)	1.8 (50)	1.2 (24)	3.5 (11)^a^	0.004*	3.9 (15)	<0.001*
Injury undetermined whether accidentally or purposely inflicted (E980-E989)	26.4 (722)	26.7 (545)	13.5 (42)	<0.001*	35.1 (135)	<0.001*
Unnatural cause of death but no E-code (ICD-9 codes 800–999)^b^	0.3 (8)	0.3 (6)	0	-	0.5 (2)	-
Other diagnosis	3	3	0	-	0	-

Opioids were set as the reference for Chi-square comparisons between the substance groups. Percentages (absolute number).

* p-value of <0.05 considered statistically significant

^a^ Fisher’s exact test used due to small sample sizes

^b^ Individuals with an unnatural cause of death but no E-code

Regarding the natural causes of death, only one significant association occurred–death by diseases of the circulatory system was less common in polysubstance group, in comparison to the opioid group (p<0.001) (S2 Table).

### Specific causes of death

Table 3 presents cases of death in which poisoning and drug or alcohol dependence or misuse were mentioned somewhere on the death certificate, including both underlying and contributing causes of death. Both accidental poisoning and poisoning with undetermined manner of death, was significantly more common in the polysubstance group, compared to the opioid group (p<0.001 respectively). Alcohol dependence syndrome as underlying or contributing cause of death was significantly less common in the polysubstance group, whereas drug dependence or misuse of drugs was significantly more common (p<0.001 respectively). Drug dependence or misuse of drugs was also more common in the stimulant group compared to the opioid group (p<0.001).

**Table 3:** Poisoning and drug or alcohol dependence or misuse, mentioned anywhere on the death certificate, according to ICD-9.

	Total, n = 2734	Opioids (and no stimulants), n = 2039	Stimulants (and no opioids), n = 310	P-value	Polysubstance group (Stimulants + opioids), n = 385	P-value
Accidental poisoning, % (n) (ICD 9-code E850-E858, E860-E869)	20.9 (571)	18.3 (374)	15.5 (48)	0.222	38.7 (149)	<0.001*
Poisoning, undetermined whether accidentally or purposely inflicted, % (n) (ICD 9-code E980)	25.4 (694)	25.6 (523)	11.6 (36)	<0.001*	35.1 (135)	<0.001*
Self-inflicted poisoning (suicide), % (n) (ICD 9-code E950)	10.8 (296)	12.9 (264)	4.8 (15)	<0.001*	4.4 (17)	<0.001*
Alcohol dependence syndrome, % (n) (ICD 9-code 303)	10.1 (275)	11.8 (240)	7.4 (23)	0.024*	3.1 (12)	<0.001*
Drug dependence or misuse, % (n) (ICD 9-code 304 or 305)	22.7 (621)	18.0 (367)	26.8 (83)	<0.001*	44.4 (171)	<0.001*

Opioids were set as the reference for Chi-square comparisons between the substance groups. Percentages (absolute number).

* p-value of <0.05 considered statistically significant

### Brain, lung and heart weights

The opioid group showed the lowest median brain weight, 1,410 grams (IQR 1,300–1,520) compared to 1,442 grams (IQR 1,346–1,536, p = 0.006) in the stimulant group and 1,461 grams (IQR 1,359–1,566, p<0.001) in the polysubstance group (Table 4). The opioid group showed the highest median heart weight, 400 grams (IQR 340–464) in comparison to 372 grams (IQR 324–425, p<0.001) in the stimulant group and 385 grams (IQR 339–448, p = 0.003) in the polysubstance group. The stimulant group had a lower median lung weight compared to the opioid group (p<0.001). The polysubstance group had a higher median lung weight (p<0.001).

**Table 4:** Brain, lung and heart weights among the study population.

	Total, n = 2734	Opioids (and no stimulants), n = 2039	Stimulants (and no opioids), n = 310	P-value	Polysubstance group (Stimulants + opioids) n = 385	P-value
Organ weights, grams, median (IQR)^a^						
Brain weight	1420 (1313–1529)	1410 (1300–1520)	1442 (1346–1536)	0.006*	1461 (1359–1566)	<0.001*
Missing^b^	2.6 (71)	2.4 (48)	2.9 (9)	-	3.6 (14)	-
Lung weight, right + left	1322 (1075–1570)	1319 (1067–1561)	1230 (967–1530)	<0.001*	1412 (1184–1636)	<0.001*
Missing^b^	0.4 (12)	0.5 (11)	-	-	0.3 (1)	-
Heart weight	395 (340–464)	400 (342–470)	372 (324–425)	<0.001*	385 (339–448)	0.003*
Missing^b^	0.3 (9)	0.4 (9)	-	-	-	-

Opioids were set as the reference value for median comparisons between the substance groups. Grams, median.

* p-value of <0.05 considered statistically significant

^a^ Mann-Whitney U test used to compare medians between groups

^b^ Proportion missing relative to the total number of participants

## Discussion

This study is based on a relatively large material of stimulant and/or opioid users, and all cases were the subjects of a forensic autopsy, which is gold standard in establishing the cause of death. As such the cause of death is not only based on findings during the autopsy, but also on circumstances surrounding the deaths. In this study, ICD codes are reported for each specific cause of death, which facilitates comparisons between studies, and has been requested in the most recent meta-analysis on mortality among amphetamine users [16].

### Characteristics, forensic and substance-related data—general

The average age of death among illicit substance users has previously been reported to be approximately 30 years [27], depending on study population and differences in treatment availability. We found a higher median age of death—45 years (Table 1). This can probably be explained by the inclusion of subjects from a forensic autopsy setting instead of subjects in a healthcare setting.

The most common drug detected besides stimulants and opioids were benzodiazepines (in 47.9% of the cases), followed by alcohol (26.0%). Co-use of both benzodiazepines and alcohol among people who use opioids or stimulants is common and is associated with an increased risk of morbidity and mortality [28–30].

### Characteristics, forensic and substance-related data

The median age at death in the polysubstance group, 34 years, is in line with previous research regarding substance users [27]. The stimulant group had a median age of death of 40 years, similar figures have been reported previously [17, 18, 20, 31], dependent on selection of participants, method of drug administration and whether all-cause mortality or only poisonings were investigated. The relatively high median age of the opioid group, 50 years, stands out. The most common cause of death in opioid users are poisoning- and substance-related causes [19], and studies on opioid-related mortality have reported a median age at death in the range of 34–39 [32, 33]. The higher median age of the opioid group in this study may be due to the group potentially including some individuals who do not have as severe opioid use disorders as in the studies reporting a lower median age, where only individuals with a history of illicit drug use are included [32, 33]. Additionally, we examined all-cause mortality and not exclusively intoxications, which likely contribute to a higher median age.

The stimulant group had a higher proportion of normal weight and a lower proportion of obese compared to the opioid group. A possible explanation for this is the known appetite-suppressing effect of stimulants that is associated with weight loss [34].

The stimulant group had a higher proportion of deaths with the simultaneous presence of THC in the blood, and a similar proportion of cases with alcohol although not significantly higher than the opioid groups. The common co-use of alcohol and cannabis among stimulant users is well established [2, 35]. In an Australian setting with amphetamine users seeking care, almost half of treatment episodes (45%) involved a client who had concurrent concern about their cannabis use and 21% involved a client with a concurrent alcohol use problem [36]. A Swedish study on patterns of drug use among subjects with illicit substance use, showed that cannabis was the most used companion substance for stimulant drug users (54%), followed by alcohol (44%) [37].

The stimulant group had a lower proportion of deaths with presence of benzodiazepines, Z-drugs and gabapentin/pregabalin compared to the opioid group. It is well documented that benzodiazepines are commonly used among people who use opioids [29, 38]. There are also data suggesting a significant use of gabapentinoids [39, 40] and Z drugs [41] among people with opioid dependence, although the number of studies is limited. There is evidence supporting that bensodiazepines and gabapentinoids could increase the rewarding and reinforcing effects of opioids [38, 42] and this could be an explanation for why these drugs are used concomitantly. Comparative studies between opioid and stimulant users with regard to polysubstance use are lacking. In an older study by Darke and Hall [43], heroin users were found to be more likely to have used benzodiazepines, while amphetamine users were more likely to have used cannabis and alcohol (as well as hallucinogens, cocaine, and inhalants). This is in line with our results, even if the difference in alcohol prevalence between the groups was not statically assured.

### Causes of death–The stimulant group

The most common cause of death in the stimulant group was suicide, 26.8%, significantly higher compared to the opioid group, 20.8%. It is slightly higher compared to what is previously reported, 6–18% [17, 18, 20, 31, 44]. A high psychiatric comorbidity among stimulant users is well established [2, 9]. In a systematic review and meta-analysis from 2019 [9] any use of amphetamines was associated with psychosis, violence, suicidality and depression. The casual pathway between use of amphetamines and these outcomes are so far unknown. We suggest longitudinal studies, controlling for several factors such as socioeconomics and comorbidity.

The stimulant group had a conclusively higher proportion of deaths in traffic accidents compared to the opioid group (15.5% and 2.8%, respectively), but also other accidents, replicating previously reported results suggesting impaired traffic-related skills in stimulant users [45, 46]. This highlights the importance of preventing risk-taking behaviors in this group.

The prevalence of homicide in the stimulant group was 3.5%, significantly higher than in the opioid group (p = 0.004). Previous studies have reported similar proportions [18, 20] and the result is in line with Eksporg and Rajs [47] who found a higher relative proportion of homicide among amphetamine users than among heroin users.

The proportion of deaths due to unclear poisoning was significantly lower in the stimulant group than in the opioid group. The assessment of an accidental or suicidal poisoning is multifactorial and rests on findings during autopsy, previous medical history and circumstances surrounding the death. To further elucidate the relevance and cause of this finding, we recommend future studies to in depth elucidate the circumstances and the medicinal history in each death and have a psychological autopsy approach.

When we analyzed to what extent substance related diagnoses appeared anywhere on the cause of death certificate (not only as an underlying cause of death), the stimulant group had a higher proportion of drug dependence and misuse (ICD 9-code 304 or 305), 26.8% compared to 18.0% in the opioid group (p<0.001). This indicate a higher proportion of individuals with a pronounced drug use problem in the stimulant group. The proportion with an alcohol dependence diagnosis appearing anywhere on the cause of death certificate was significantly lower than in the opioid group. This result is somewhat surprising, both considering how common alcohol is among stimulant users [2, 35], and the tendency for a higher percentage of individuals with alcohol in their blood at death in the stimulant group (although the difference compared to the opioid group was not statistically significant). One can speculate whether differing practices among forensic pathologists in coding of such contributing causes of death can be an explanation.

### Causes of death—The polysubstance group

The polysubstance group had a significantly higher proportion of unnatural deaths overall compared to the opioid group. The most common cause of death was accidental poisoning, 38.2% died from it–a significantly higher percentage than the opioid group’s 18% (p<0.001). Concomitant use of opioids and stimulants has been shown to be a risk factor for overdose, compared to only opioids [48]. Moreover, the combination of opioids and benzodiazepines is known to particularly increase the risk of fatal overdose [38] and benzodiazepines occurred to a greater extent in the polysubstance group, 64.2% vs 46.9% in the opioid group (p<0.001) (Table 1), which also could contribute to the higher proportion of accidental poisoning.

The polysubstance group also had a significantly higher proportion of individuals who died from injury with unclear intent, 35.1%, in comparison to 26.7% in the opioid group (p<0.001) (Table 2). A possible explanation is the higher proportion of people with known substance use, among whom there seems to be a greater uncertainty about the intent of the deaths [49]. Finally, in the polysubstance group there may be more cases with several low post-mortem substance concentrations, which in combination with a lack of information on the individuals mental state makes it difficult for the forensic pathologists to establish the intent of the event.

The polysubstance group also had a significantly higher proportion of homicides, 3.9% compared to the opioid group’s 1.2% (p<0.001), this strengthens the image that the polysubstance group contains more individuals with a pronounced substance use problem who thus expose themselves to greater risks of violence [27].

When we analyzed to what extent poisoning and substance related diagnoses appeared anywhere on the cause of death certificate, the polysubstance group was found to have a higher proportion of poisonings than the opioid group, both accidental poisonings and poisonings with unclear intent. The proportion with drug dependence or misuse (ICD 9-code 304 or 305) somewhere on the cause of death certificate was 44.4% in the polysubstance group, significantly higher than the opioid group’s proportion of 18.0% (p<0.001) (Table 3). This is in line with the conclusion earlier, that the polysubstance group contains more individuals with a pronounced drug use problem.

### Brain, lung and heart weights

We examined organ weights that alone are of interest in the setting of acute or chronic drug intake, as such the lungs and brain are of interest [50], and in the perspective of chronic disease, the heart is of interest [3–5, 21].

Both the stimulant group and the polysubstance group had higher median brain weight than the opioid group. One explanation in the polysubstance group, is the higher proportion of poisoning deaths where cerebral edema is a common finding [50]. The differences in brain weight are however numerically small in both the stimulant and the polysubstance group and should therefore be interpreted with caution. The stimulant and the polysubstance group had lower median heart weight than the opioid group. Both groups also had significantly lower median age at death compared to the opioid group, and there are some evidence suggesting heart weight to increase with age [51], however, there is also conflicting results [52]. The individual variation in organ weights is evident, depending on several different factors such as the weight and diseases of the individual [52]. Furthermore, the results are not adjusted for age. Overall, the results here should be interpreted with caution.

The polysubstance group had higher median lung weight than the opioid group. Previous studies have suggested that increased lung weight could be associated with opioid poisonings, with pulmonary edema as a common finding [50, 53, 54]. However, more recent studies have shown that increased lung weight is rather associated with poisonings in general [55, 56] and not specifically poisonings with opioids. The mechanism of fatal opioid poisoning is respiratory depression, a process enhanced by the co-intoxication of other drugs [54]. The difference in lung weight in our case may thus be an expression of the higher proportion of poisoning deaths in the polysubstance group.

### Implications

Forming a deeper understanding of the mortality and deaths of people who use stimulants is critical to the development and provision of effective treatment and harm reduction interventions for this group. The results of this study indicate that there is reason to pay more attention in the clinical setting to the risk of accidents, and in particular traffic-related risks, in stimulant users. Another important clinical implication is to better assess and follow up the suicide risk among people who use stimulants. In the forensic setting, the study highlights the importance of adherence to standards for unbiased assessments of cause and manners of death during a forensic autopsy.

### Limitations and strengths

There is no international agreement on how drug-related mortality should be defined. Definitions vary between countries, and each definition has its limitations [57]. There is a wide range of plausible mechanisms between substance use and death [58, 59]. Acute poisoning, chronic organ damage caused by drugs as well as accidents during intoxication may all be considered to be drug-related. In this study, we wanted to investigate a more unselected population of substance users, not only users who received a substance-related diagnosis. We therefore identified cases based on toxicological findings in forensic autopsies. We consider every death in which opioids or stimulants were detected during postmortem toxicology analysis as potentially related to the use of these substances.

The present study has limitations. First, the results cannot be interpreted as causal relationships. Furthermore, we have no information on the length of the individuals’ drug careers. It is possible that the material contains some individuals who lack a substance use problem. For example, in the opioid group there may be older individuals who used morphine for pain and in the stimulant group there may be individuals with ADHD who used methylphenidate. Nevertheless, most of our results do not differ considerably from studies based on material from individuals with diagnosed substance use problem, which to some extent validates the included population.

Since we used substances detected in forensic toxicological analysis as a method to identify groups of substance users one should bear in mind the different pharmacokinetics of the substances, which affects the ability to detect the substance in the toxicological analysis. In general, opioids and stimulants can be detected a few days after the last intake, while THC and benzodiazepines, which have longer half-time, can be detected up to weeks after the last intake [60]. Thus, stimulant and opioid users who did not use the substance in question within this time frame prior to their death have not been identified as a user in the study. However, it is reasonable to assume that users with a regular use of these substances are included.

Another limitation is that we were unable to investigate whether the individuals in the study were prescribed the substances that were found during toxicological analysis. However, even if data on prescribed medications were available, we would still be unable to state whether the medication was used as prescribed. Furthermore, we lack information on the individuals’ medical history such as psychiatric and somatic disorders. However, the causes of death determined by the forensic pathologist could reflect pre-existing pathological conditions that were considered to contribute to the death and would, in that sense, be a part of the study’s result. To further assess co-morbidities in the different groups is beyond the scope of this study. Future studies should though include additional data from other authorities and institutions to obtain in-depth knowledge of the group differences demonstrated in this study. This could for example include information from the social service on previous contacts as well as information from medical records or registers regarding diagnoses and treatments.

Finally, in this study, we did not have access to tissue samples, which could have contributed with additional knowledge on chronic organ damage among opioid and stimulant users. Future studies may benefit from including such data.

This study consists of a relatively large material of individuals who died with the presence of stimulants or/and opioids in their body and covers a complete regional population. All individuals in the study underwent a forensic autopsy, which enables a reliable determination of the cause of death. The study contains details on causes and manners of death including specific ICD codes, among the stimulant group in comparison to the opioid group.

## Conclusions

Individuals who died with the presence of stimulants in their body in this forensic material died at a higher rate from suicide, transport accidents and other accidents, compared to individuals who died with the presence of opioids in their body. Concomitant use of other substances was common across the groups. The combination of stimulants and opioids appears to be correlated with accidental overdose death.

This study indicates the need to identify and prevent psychiatric conditions and elevated suicide risk, risk-taking behaviors and polysubstance use among people who use stimulants. However, more studies are needed, that for example includes anamnestic data, to further assess the group differences demonstrated in this study, and to better understand the specific characteristics and risks among stimulant-using individuals.

## Supporting information

S1 Table Methods used to analyze the included substances. (DOCX)

S2 Table Natural causes of death according to ICD-9, specified. (DOCX)
