# Supplementary material for: Causes and circumstances of death in stimulant and opioid use—A comparative study
Source: PLoS One. 2024 Feb 7;19(2):e0297838. doi: 10.1371/journal.pone.0297838 (PMC10849257; doi:10.1371/journal.pone.0297838)
Supplement: S1 Table — (DOCX) [file pone.0297838.s001.docx]

**S1 Table. Methods used to analyze the included substances**

All toxicological analyses were performed between 2000-2018 at the same national laboratory - the Division for Forensic Toxicology in Linköping, Sweden.

| **Substance** | **Method** |
| --- | --- |
| Ethanol | GC-FID |
| Heroine (6MAM), morphine | GC-MS |
| Methadone, Tramadol | GC-NPD 2000-2012, > June 2012 LC-MSMS |
| Buprenorfin | routinely analysed since 2010 LS-MSMS |
| Oxycodone | LC-MSMS |
| Phentanyl (Phentanylanalogues) | LC-MSMS |
| Amphetamine, methamphetamin, MDMA | GC-MS |
| Methylphenidate | LC-MSMS |
| Modafinil | LC-MSMS |
| Cocaine | GC-MS 2000-2018, > Oct 2018 LC-MSMS |
| Bensodiazepines | GC-NPD 2000-2011, > Oct 2011 LC-MSMS |
| THC | GC-MS |
