# Supplementary material for: Causes and circumstances of death in stimulant and opioid use—A comparative study
Source: PLoS One. 2024 Feb 7;19(2):e0297838. doi: 10.1371/journal.pone.0297838 (PMC10849257; doi:10.1371/journal.pone.0297838)
Supplement: S2 Table — (DOCX) [file pone.0297838.s002.docx]

**S2 Table. Natural causes of death according to ICD-9, specified.**

|  | Total,  n=2734 | Opioids  (and no stimulants), n=2039 | Stimulants  (and no opioids), n=310 | P-value | Polysubstance group  (Stimulants + opioids), n=385 | P-value |
| --- | --- | --- | --- | --- | --- | --- |
| Natural causes of death, % (n) | 22.5 (615) | 25.4 (518) | 21.0 (65) | 0.092 | 8.3 (32) | <0.001* |
| Neoplasms  (ICD-9 codes 140–239) | 0.9 (24) | 1.2 (24) | 0^a^ | 0.063 | 0^a^ | 0.024* |
| Endocrine, nutritional and metabolic diseases, and immunity disorders  (ICD-9 codes 240–279) | 0.7 (19) | 0.8 (16) | 0.6 (2)^a^ | 1.000 | 0.3 (1)^a^ | 0.500 |
| Mental disorders  (ICD-9 codes 290–319) | 1.3 (36) | 1.2 (25) | 2.3 (7)^a^ | 0.181 | 1.0 (4)^a^ | 1.000 |
| Diseases of the nervous system and sense organs  (ICD-9 codes 320–389) | 0.4 (10) | 0.3 (6) | 1.0 (3)^a^ | 0.104 | 0.3 (1)^a^ | 1.000 |
| Diseases of the circulatory system  (ICD-9 codes 390–459) | 13.9 (380) | 16.0 (326) | 12.6 (39) | 0.123 | 3.9 (15) | <0.001* |
| Diseases of the respiratory system  (ICD-9 codes 460–519) | 2.9 (78) | 3.0 (62) | 2.9 (9) | 0.895 | 1.8 (7) | 0.186 |
| Diseases of the digestive system  (ICD-9 codes 520–579) | 1.3 (36) | 1.6 (32) | 0.6 (2)^a^ | 0.305 | 0.5 (2)^a^ | 0.153 |
| Symptoms, signs, and ill-defined conditions  (ICD-9 codes 780–799) | 0.9 (25) | 1.0 (21) | 1.0 (3)^a^ | 1.000 | 0.3 (1)^a^ | 0.236 |
| Other diagnosis | 7 | 6 | 0 | - | 1 | - |

Opioids were set as the reference for Chi-square comparisons between the substance groups. Percentages.

* p-value of <0.05 considered statistically significant

^a^ Fisher’s exact test used due to small sample sizes
